# Distribution and genetic diversity of *Anisakis* spp. in cetaceans from the Northeast Atlantic Ocean and the Mediterranean Sea

**DOI:** 10.1038/s41598-022-17710-1

**Published:** 2022-08-11

**Authors:** Paolo Cipriani, Marialetizia Palomba, Lucilla Giulietti, Federica Marcer, Sandro Mazzariol, Mario Santoro, Renato Aco Alburqueque, Pablo Covelo, Alfredo López, M. Begoña Santos, Graham J. Pierce, Andrew Brownlow, Nicholas J. Davison, Barry McGovern, Alexandros Frantzis, Paraskevi Alexiadou, Dánjal Petur Højgaard, Bjarni Mikkelsen, Michela Paoletti, Giuseppe Nascetti, Arne Levsen, Simonetta Mattiucci

**Affiliations:** 1 Department of Public Health and Infectious Diseases, Section of Parasitology, Sapienza University of Rome, Rome, Italy; 2 Institute of Marine Research (IMR), Nordnes, Bergen, Norway; 3 Department of Ecological and Biological Sciences (DEB), Tuscia University, Viterbo, Italy; 4 Department of Animal Medicine, Production and Health, Padova University, Padova, Italy; 5 Department of Integrative Marine Ecology, Stazione Zoologica Anton Dohrn, Naples, Italy; 6 Coordinadora para o Estudo dos Mamíferos Mariños CEMMA, Gondomar, Pontevedra Spain; 7 Departamento de Biología & CESAM, Universidade de Aveiro, Aveiro, Portugal; 8 Instituto Español de Oceanografía, Centro Oceanográfico de Vigo, Vigo, Spain; 9 Instituto de Investigaciones Marinas (CSIC), Vigo, Spain; 10 Scottish Marine Animal Scheme, Institute of Biodiversity, Animal Health & Comparative Medicine, University of Glasgow, Glasgow, UK; 11 Pacific Whale Foundation Australia, Urangan, Australia; 12 Pelagos Cetacean Research Institute, Vouliagmeni, Greece; 13 Faroe Marine Research Institute (Havstovan), Tórshavn, Faroe Islands

**Keywords:** Biodiversity, Parasitology

## Abstract

Parasite biodiversity in cetaceans represents a neglected component of the marine ecosystem. This study aimed to investigate the distribution and genetic diversity of anisakid nematodes of the genus *Anisakis* sampled in cetaceans from the Northeast Atlantic Ocean and the Mediterranean Sea. A total of 478 adults and pre-adults of *Anisakis* spp. was identified by a multilocus genetic approach (mtDNA *cox*2*,* EF1 *α* − 1 nDNA and *nas* 10 nDNA gene loci) from 11 cetacean species. A clear pattern of host preference was observed for *Anisakis* spp. at cetacean family level: *A. simplex* (s.s.) and *A. pegreffii* infected mainly delphinids; *A. physeteris* and *A. brevispiculata* were present only in physeterids, and *A. ziphidarum* occurred in ziphiids. The role of cetacean host populations from different waters in shaping the population genetic structure of *A. simplex* (s.s.), *A. pegreffii* and *A. physeteris* was investigated for the first time. Significant genetic sub-structuring was found in *A. simplex* (s.s.) populations of the Norwegian Sea and the North Sea compared to those of the Iberian Atlantic, as well as in *A. pegreffii* populations of the Adriatic and the Tyrrhenian Seas compared to those of the Iberian Atlantic waters. Substantial genetic homogeneity was detected in the Mediterranean Sea population of *A. physeteris.* This study highlights a strong preference by some *Anisakis* spp. for certain cetacean species or families. Information about anisakid biodiversity in their cetacean definitive hosts, which are apex predators of marine ecosystems, acquires particular importance for conservation measures in the context of global climate change phenomena.

## Introduction

Anisakid nematodes of the genus *Anisakis* Dujardin, 1845 are marine parasites with a worldwide distribution. Their life cycle is indirect, involving several hosts at different trophic levels of marine food webs. The parasite’s adult stage lives in marine mammals, mainly cetaceans, while planktonic or semi-planktonic crustaceans act as first intermediate hosts of the parasite, and fish and squid represent intermediate/paratenic hosts^1–3^. Specifically, *Anisakis* spp. parasitize the digestive tracts of cetaceans, being commonly found in the stomachs of both toothed and baleen whales^1,3–7^. The definitive hosts acquire *Anisakis* through ingestion of infected crustaceans, fish or squid. Once in the stomach of cetacean hosts, the third larval stages (L3) of *Anisakis* spp. moult into the fourth stage larvae (L4), that further develop into sexually mature adults, a process taking from 40 to 60 days^8–10^. The adult stages of *Anisakis* “swim” in the ingesta, probably feeding on them^9^. In many cases they attach themselves to the stomach mucosa, forming clusters and sometimes generating gastric granulomatous ulcers^11–15^.

The application of genetic/molecular methodologies to *Anisakis* morphospecies has advanced our understanding of their systematics, taxonomy, and phylogeny, allowing the detection and description of sibling species and the discovery of new species^2,3,16–18^, and generally furthering our knowledge of their ecology, geographical distribution and host preferences^1,3,19–22^. The existence of nine nominal species of *Anisakis* as distinct phylogenetic units has been demonstrated by various phylogenetic analyses, using both nuclear and mitochondrial genes^2,3,19,23,24^. PCR–RFLP of the ITS rDNA region^24^ and the sequencing of the mitochondrial mtDNA *cox2* gene^25^ are used to identify all the species of *Anisakis,* at both larval and adult stages. Several nuclear gene loci have been recently validated on specimens of the *A. simplex* s.l. complex (i.e. *A. pegreffii*, *A. simplex* (s.s.) and *A. berlandi*): sequence analysis of the EF1 α − 1 nDNA region revealed two diagnostic nuclear sites (SNPs) at which the nucleotides differed between *A. simplex* (s.s.) and *A. pegreffii*^26^; the metallopeptidase 10 (*nas*10 nDNA) locus exhibits diagnostic SNPs, at which the nucleotides differ between the three species, and for which ARMS-PCR assays were developed^27^. Panels of DNA microsatellite (SSR) loci have been developed in recent years^17,28^, which include some 100% diagnostic loci with fixed alternative alleles which differ between the three species^17,29,30^.

Studies on the epidemiology of *Anisakis* spp. in their intermediate/paratenic hosts (i.e. fish and squid) from Northeast (NE) Atlantic Ocean and Mediterranean Sea are abundant, and this field is regularly updated^3,31–35^. However, there is scant epidemiological data on *Anisakis* spp. in their definitive hosts (i.e., cetaceans) and consequently knowledge is lacking on their biodiversity, distribution, and ecology at the pre-adult and adult stages. To date, investigations on parasites of cetaceans in the NE Atlantic and Mediterranean Sea have relied on samples from stranded individuals^3,12,14,15,36–39^ or, more rarely, on samples obtained during whaling activities^8^.

Knowledge of the biodiversity (at both species and genus level) of *Anisakis* in different cetacean hosts has important implications for the understanding of the parasite/host ecology. The aim of the present work was to apply a multilocus genetic approach to the study of anisakid endoparasites of cetaceans from European seas, to: (i) assess the biodiversity of *Anisakis* species at their adult stage in various cetacean hosts, from different areas of the NE Atlantic Ocean and the Mediterranean Sea; (ii) provide insights into the distribution of *Anisakis* species in cetacean populations; (iii) analyse the host-parasite association between cetacean hosts and their *Anisakis* parasites; (iv) investigate the population genetic structure of *Anisakis* spp. from different hosts and geographical ranges.

## Results

### Parasite samples

A total of N = 478 anisakid nematodes was obtained from stranded cetaceans and, according to their morphological characteristics, they were all assigned to the *Anisakis* genus. Only adult (showing developed reproductive structures, such as caudal papillae and spicules in male worms and ovaries in females) and pre-adult (presence of labia, absence of boring tooth) specimens were found. It was not possible to count or estimate the total burden of worms during most of the necropsies of cetaceans, only to obtain a subsample of parasites. Hence, we could not perform a comprehensive epidemiological analysis of the parasites for each host species.

### Genetic identification of *Anisakis*

**Figure 1:**
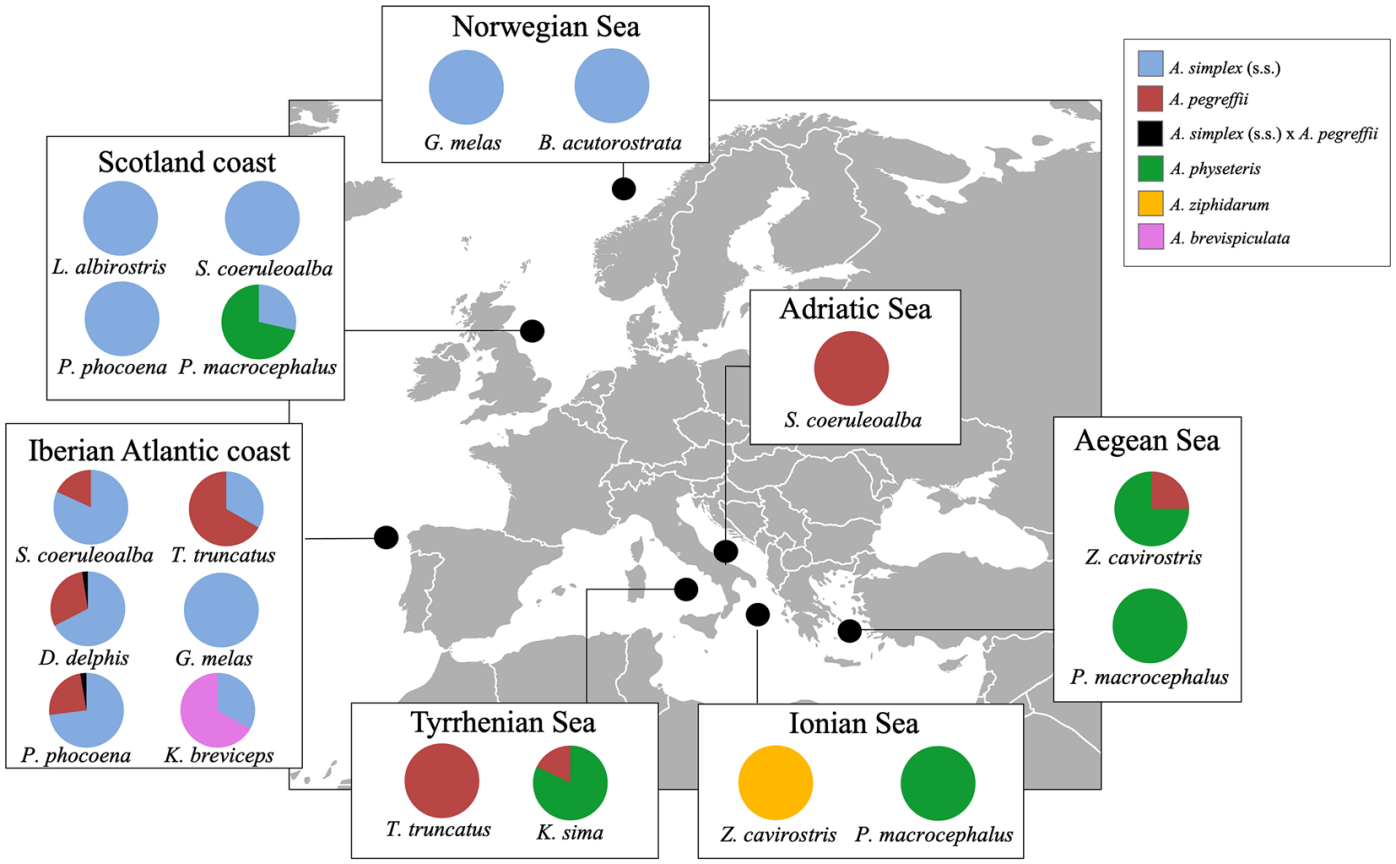
Relative proportions of *Anisakis* spp. in cetaceans according to different geographical areas of the NE Atlantic Ocean and the Mediterranean Sea. The map was obtained from Wikimedia Commons, licensed-free (https://commons.wikimedia.org/wiki/File:BlankMap-Europe-v4.png), Roke was the unofficial uploader of the derivitive map CC BY-SA 3.0.

**Figure 2:**
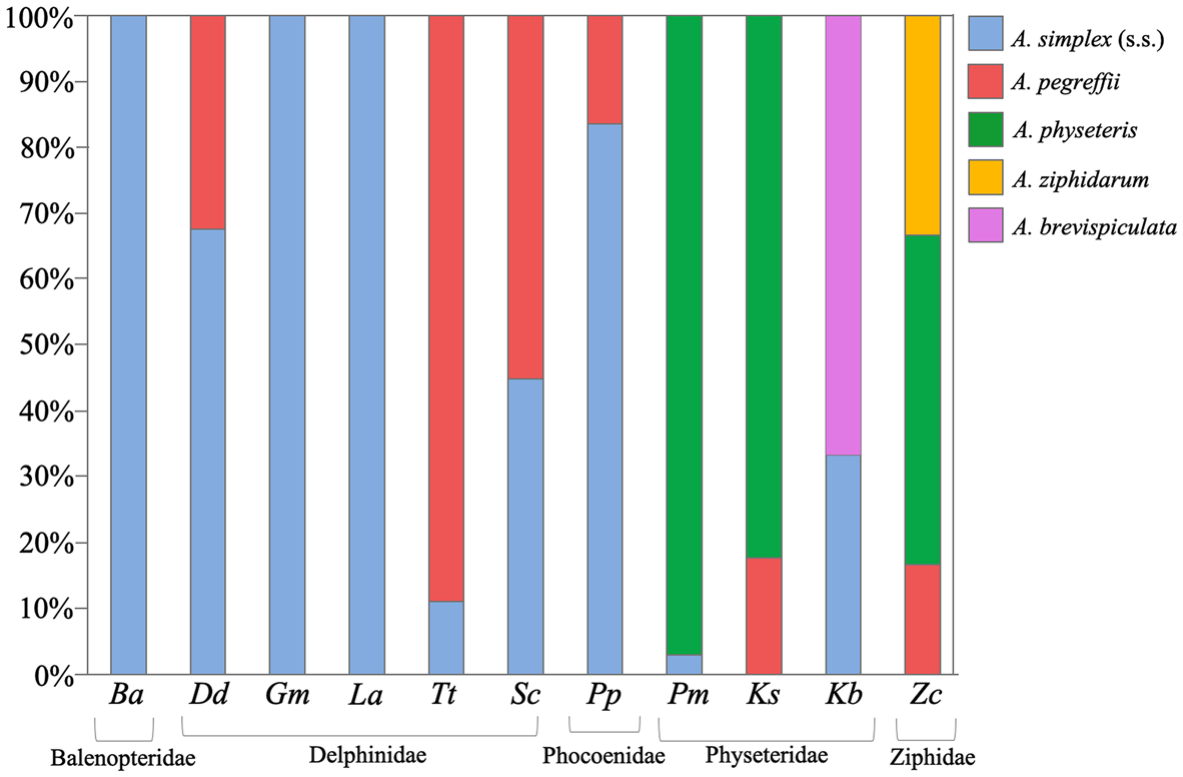
Relative proportions of *Anisakis* spp. according to definitive hosts belonging to species of families Balenopteridae, Delphinidae, Phocoenidae, Physeteridae and Ziphiidae. Host codes are reported by alphabetical order: *Ba*: *Balenoptera acutorostrata*, *Dd*: *Delphinus delphis*, *Gm*: *Globicephala melas*, *La*: *Lagenorhynchus albirostris*, *Tt*: *Tursiops truncatus*, *Sc*: *Stenella coeruleoalba*, *Pp*: *Phocoena phocoena*, *Pm*: *Physeter macrocephalus*, *Ks*: *Kogia sima*, *Kb*: *Kogia breviceps*, *Zc*: *Ziphius cavirostris*.

According to the BLAST analysis performed on the 478 mtDNA *cox*2 sequences (580 bp) obtained, five species were detected: *A. simplex* (s.s.), *A. pegreffii, A. physeteris, A. brevispiculata* and *A. ziphidarum*. The numbers of *Anisakis* specimens genetically identified in the definitive hosts from different sampling area are reported in Table 1. The relative proportions of *Anisakis* spp. in cetaceans in different geographical areas of the NE Atlantic Ocean and the Mediterranean Sea, and in their different definitive hosts families, are reported in Figs. 1 and 2, respectively.

**Table 1:** Number (N_I_) of *Anisakis* specimens genetically identified in the definitive hosts from different sampling area.

Host family	Cetacean species	Sampling area	N_I_	*A. simplex* (s.s.)	*A. pegreffii*	*A.simpl/A.peg* Heterozygote	*A. physeteris*	*A. brevispiculata*	*A. ziphidarum*
**Mysticeti**									
Balenopteridae	*Balenoptera acutorostrata*	Norwegian Sea	25	25	–	–	–	–	–
**Physeteroidea**									
Physeteridae	*Physeter macrocephalus*	Scotland coast	7	2	–	–	5	–	–
		Adriatic Sea	60	–	–	–	60	–	–
		Aegean Sea	1	–	–	–	1	–	–
Kogiidae	*Kogia breviceps*	Iberian Atlantic coast	3	1	–	–	–	2	–
	*Kogia sima*	Tyrrhenian Sea	107	–	19	–	88	–	–
**Ziphiidae**									
Ziphiidae	*Ziphius cavirostris*	Aegean Sea	4	–	1	–	3	–	–
		Ionian Sea	2	–	–	–	–	–	2
**Delphinoidea**									
Delphinidae	*Delphinus delphis*	Iberian Atlantic coast	34	23	10	1	–	–	–
	*Lagenorhynchus albirostris*	Scotland coast	22	22	–	–	–	–	–
	*Stenella coeruleoalba*	Scotland coast	21	21	–	–	–	–	–
		Iberian Atlantic coast	22	18	4	–	–	–	–
		Adriatic Sea	44	–	44	–	–	–	–
	*Tursiops truncatus*	Iberian Atlantic coast	3	1	2	–	–	–	–
		Tyrrhenian Sea	6	–	6	–	–	–	–
	*Globicephala melas*	Faroe Islands	34	34	–	–	–	–	–
		Iberian Atlantic coast	27	27	–	–	–	–	–
Phocoenidae	*Phocoena phocoena*	Scotland coast	9	9	–	–	–	–	–
		Iberian Atlantic coast	47	37	9	1	–	–	–
		*Tot.*	478	220	96	2	157	2	2

The sympatric (in some cases even syntopic, such as in two specimens of common dolphins *Delphinus delphis*, one striped dolphin *Stenella coeruleoalba*, one bottlenose dolphin *Tursiops truncatus* and three harbour porpoises *Phocoena phocoena*) occurrence of 134 adult specimens of *A. pegreffii* and *A. simplex* (s.s.) was recorded from dolphin species stranded along the Iberian Atlantic coast (Table 1). These specimens were also identified using nuclear diagnostic loci, i.e. the EF1 *α* − 1 nDNA and *nas*10 nDNA loci. Among them, 134 specimens showed 100% concordance with the mtDNA *cox*2 data set for the identification of the two parental taxa, i.e. *A. simplex* (s.s.) (N = 107) and *A. pegreffii* (N = 27). Indeed, at the EF *α − *1 nDNA locus, 107 individuals were found to be homozygous C/C and T/T, respectively, at the fixed alternative nucleotide positions 186 and 286, i.e. diagnostic for the parental species *A. simplex* (s.s.). A further 25 showed the homozygote genotypes T/T and C/C at the same SNPs, corresponding to the parental species *A. pegreffii*^26^ (Suppl. Fig. 1). Additionally, the ARMS-PCR analysis enabled us to genotype 107 individuals as belonging to *A. simplex* (s.s.), and 25 to the species *A. pegreffii*. The combined use of the *nas* 10 primers generated a specific PCR product of 296 bp in *A. simplex* (s.s.), amplifying the T-allele, and a specific PCR fragment of 117 bp in *A. pegreffii*, amplifying the C-allele. In contrast, two specimens obtained respectively from a harbour porpoise and a short-beaked common dolphin, which at the mtDNA *cox*2 locus corresponded to the species *A. pegreffii,* showed a heterozygote pattern at those diagnostic positions (SNPs)—thus falling between *A. pegreffii* and *A. simplex* (s.s.), in both the two nuclear loci EF1 *α* − 1 nDNA and *nas*10 nDNA (Suppl. Fig. 1). In particular, the sequence analysis of these two specimens revealed the occurrence of a double peak at these diagnostic positions (i.e., 186 and 286) at the EF1 *α* − 1^26^. For the ARMS analysis, two specific PCR fragments of both 296 and 117 bp were generated in the species with a heterozygote pattern, amplifying both the C and T allele. This latter finding showed 100% concordance with the direct sequence analysis of the gene locus *nas* 10 nDNA. These two specimens, showing a heterozygous pattern at all the diagnostic SNPs of the EF1 *α* − 1 and *nas*10 nDNA loci, seem to be the result of hybridization events between *A. pegreffii* and *A. simplex* (s.s.).

The DNA sequences of the presently identified *Anisakis* species were deposited in GenBank under the following accession numbers: mtDNA *cox*2: OM142467–OM142471 (*A. pegreffii*); OM142472–OM142476 (*A. simplex* (s.s.)); OM14247–OM142481 (*A. physeteris*). EF1 *α* − 1 nDNA: OM210033–OM210037 (*A. pegreffii*)*;* OM210038 (*A. simplex* (s.s.)).

### Phylogenetic analysis inferred from mtDNA *cox*2 gene locus

**Figure 3:**
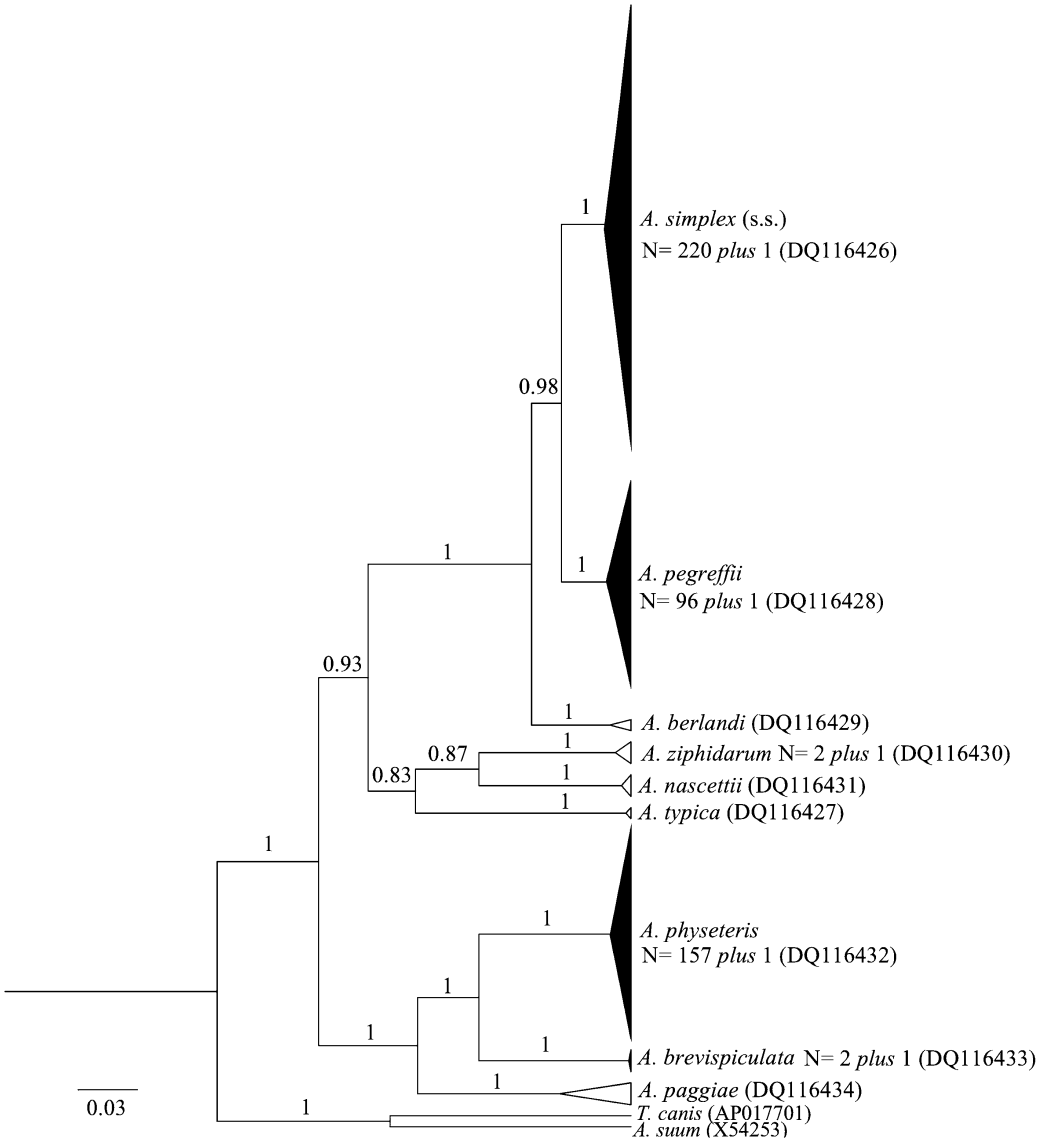
Phylogenetic tree of *cox*2 mtDNA gene locus constructed using Bayesian Markov Chain Monte Carlo (MCMC) analysis in BEAST v1.10.4^109^. GTR + I + G substitution model, strict molecular clock^103^ and Yule speciation process were used as the tree priors, Bayesian inference (BI) on mtDNA *cox*2 gene sequences of *Anisakis* spp. obtained from cetaceans. *Toxocara canis* and *Ascaris suum* were used as an outgroup.

The Bayesian inference (BI) phylogenetic tree based on mtDNA *cox*2 sequences (Fig. 3) indicated that a total of 220 nematodes, including preadult and adult specimens recovered in minke whale (*Balaenoptera acutorostrata*) and six delphinid species (striped dolphin *Stenella coeruleoalba*, bottlenose dolphin *Tursiops truncatus*, harbour porpoises *Phocoena phocoena,* long-finned pilot whale *Globicephala melas,* white-beaked dolphin *Lagenorhynchus albirostris*, and common dolphin *Delphinus delphis*) and a few preadults found in physeterids (sperm whale *Physeter macrocephalus* and pygmy sperm whale *Kogia breviceps*), clustered together within a well-supported clade (100% probability value), together with a reference sequence of *A. simplex* (s.s.) (DQ116426) (Fig. 3). Similarly, a total of 96 *Anisakis* preadult and adult specimens grouped in a highly (100%) supported phylogenetic lineage represented by *A. pegreffii*, which also included a reference sequence (DQ116428) of the parasite species (Fig. 3). Additionally, 157 specimens collected in physeterids from the Mediterranean Sea and the Atlantic Ocean clustered in a well-supported distinct phylogenetic clade formed by the species *A. physeteris* (Fig. 3). Two adult *Anisakis* specimens collected from pygmy sperm whale (*Kogia breviceps*) in Atlantic waters clustered in a clade represented by *A. brevispiculata* (Fig. 3, Table 1). Two adult worms collected from Cuvier's beaked whale (*Ziphius cavirostris*) from the Aegean Sea were placed in a highly supported clade comprising sequences of *A. ziphidarum* previously deposited in GenBank (Fig. 3, Table 1).

### Genetic diversity inferred from mtDNA *cox*2 gene locus

The genetic diversity analysis was performed at intraspecific level in *A. simplex* (s.s.), *A. pegreffii* and *A. physeteris*. It was not possible to give estimates of genetic diversity of *A. ziphidarum* and *A. brevispiculata* because of the low number of worms identified for those species.

The hierarchical gene diversity analysis by AMOVA (Suppl. Table 1) indicated that in *A. simplex* (s.s.) 92.77% of the genetic variation was expressed within populations and only 2.52% was variation among populations of this parasite species collected from cetacean hosts of different geographic areas of the NE Atlantic Ocean (Suppl. Table 1). Similar results were obtained for *A. pegreffii,* with 97.48% of variation occurring within populations of the parasite species collected in cetaceans from different areas (Suppl. Table 1). In the case of *A. physeteris* 94.62% of variation occurred within populations (Suppl. Table 1).

Estimates of the genetic differentiation (*F*_*st*_) between populations of the presently identified *Anisakis* species, as inferred from the fixation index, are shown in Table 2. At the intraspecific level, a significant genetic differentiation (*F*_*st*_) was found between *A. simplex* (s.s.) metapopulations from the Norwegian Sea compared to those from Iberian Atlantic coast (*F*_*st*_ ≈ 0.12, *p* < 0.00001), as well as between populations of the same species from the Scottish and Iberian Atlantic coast (*F*_*st*_ ≈ 0.07, *p* < 0.00001). However, *A. simplex* (s.s.) individuals collected from the Norwegian Sea had a lower pairwise *F*_*st*_ when compared to its conspecifics in Scottish waters (*F*_*st*_ ≈ 0.02, *p* = 0.03) (Table 2). Comparing host species, significant *F*_*st*_ values were found between *A. simplex* (s.s.) metapopulations from long-finned pilot whales (Faroes Islands and Iberian Atlantic) and those collected from the minke whale from Norwegian Sea (*F*_*st*_ ≈ 0.10, *p* < 0.00001) and white-beaked dolphin from Scottish waters (*F*_*st*_ ≈ 0.07, *p* < 0.00001) (data not shown). However, a higher *F*_*st*_ pairwise value (*F*_*st*_ ≈ 0.15, *p* < 0.001) was recorded when comparing parasite metapopulations from the same cetacean species, i.e. pilot whale subpopulation from Faroes Island and Iberian Atlantic coast. Similarly, a significant genetic differentiation was found when comparing the metapopulations of *A. simplex* (s.s.) from striped dolphins of Iberian Atlantic coast and Scottish waters (*F*_*st*_ ≈ 0.09, *p* < 0.00001).

**Table 2:** Population pairwise F_ST_ estimates inferred from mtDNA *cox*2 sequences analysis among *A. simplex* (s.s.) (a) and *A. pegreffii* (b) and *A. physeteris* (c) collected from cetacean of different localities.

	Iberian Atlantic coast	Adriatic Sea	Tyrrhenian Sea
**a. *A. pegreffii***			
Iberian Atlantic coast	–	0.00000 ± 0.0000	0.01465 ± 0.0039
Adriatic Sea	0.07006	–	0.10449 ± 0.0081
Tyrrhenian Sea	0.04070	0.01569	–

	Iberian Atlantic coast	Scottish coast	Norwegian Sea
**b. *A. simplex* (s.s.)**			
Iberian Atlantic coast	–	0.00000 ± 0.0000	0.00000 ± 0.0000
Scottish coast	0.07611	–	0.02637 ± 0.0061
Norwegian Sea	0.12307	0.02101	–

	Ionian Sea	Tyrrhenian Sea	Aegean Sea
**c. *A. physeteris***			
Ionian Sea	–	0.03418 ± 0.0056	0.03809 ± 0.0044
Tyrrhenian Sea	0.01228	–	0.13574 ± 0.0094
Aegean Sea	0.12020	0.05631	–

Below the diagonal: conventional F_ST_ from haplotype frequencies; above the diagonal: *p* values of F_ST_.

Intraspecific genetic variation between metapopulations of *A. pegreffii* from the Adriatic and the Tyrrhenian Seas was not significant (*F*_*st*_ ≈ 0.015, *p* = 0.10) (Table 2). In contrast, significant genetic sub-structuring was observed when comparing the Mediterranean (Adriatic and Tyrrhenian) populations of *A. pegreffii* with those from the Iberian Atlantic coast (*F*_*st*_ ≈ 0.07 (*p* = 0.0001) and *F*_*st*_ ≈ 0.04 (*p* = 0.001)*,* respectively (Table 2)). Due to the low number of *A. pegreffii* found in the different definitive hosts examined, except for striped dolphins, it was not possible to compare the *Fst* value at intraspecific level by host species.

Due to the low number of specimens of *A. physeteris* obtained from the NE Atlantic Ocean, *F*_*st*_ at the intraspecific level was calculated only for the comparison of specimens collected from stranded sperm whales along the coast of the Adriatic Sea (of Ionian Sea origin^31^) and those carried by the dwarf sperm whale (*Kogia sima*) from the Tyrrhenian Sea, with the finding of a generally low and slightly significant level of differentiation (*F*_*st*_ ≈ 0.012, *p* = 0.03) (Table 2).

At the interspecific level, significant values of genetic differentiation were observed between *A. simplex* (s.s.) and *A. pegreffii* (*F*_*st*_ ≈ 0.836), with the highest values being those found between *A. physeteris* and *A. simplex* (s.s.) (*F*_*st*_ ≈ 0.945) and between *A. physeteris* and *A. pegreffii* (*F*_*st*_ ≈ 0.940).

**Figure 4:**
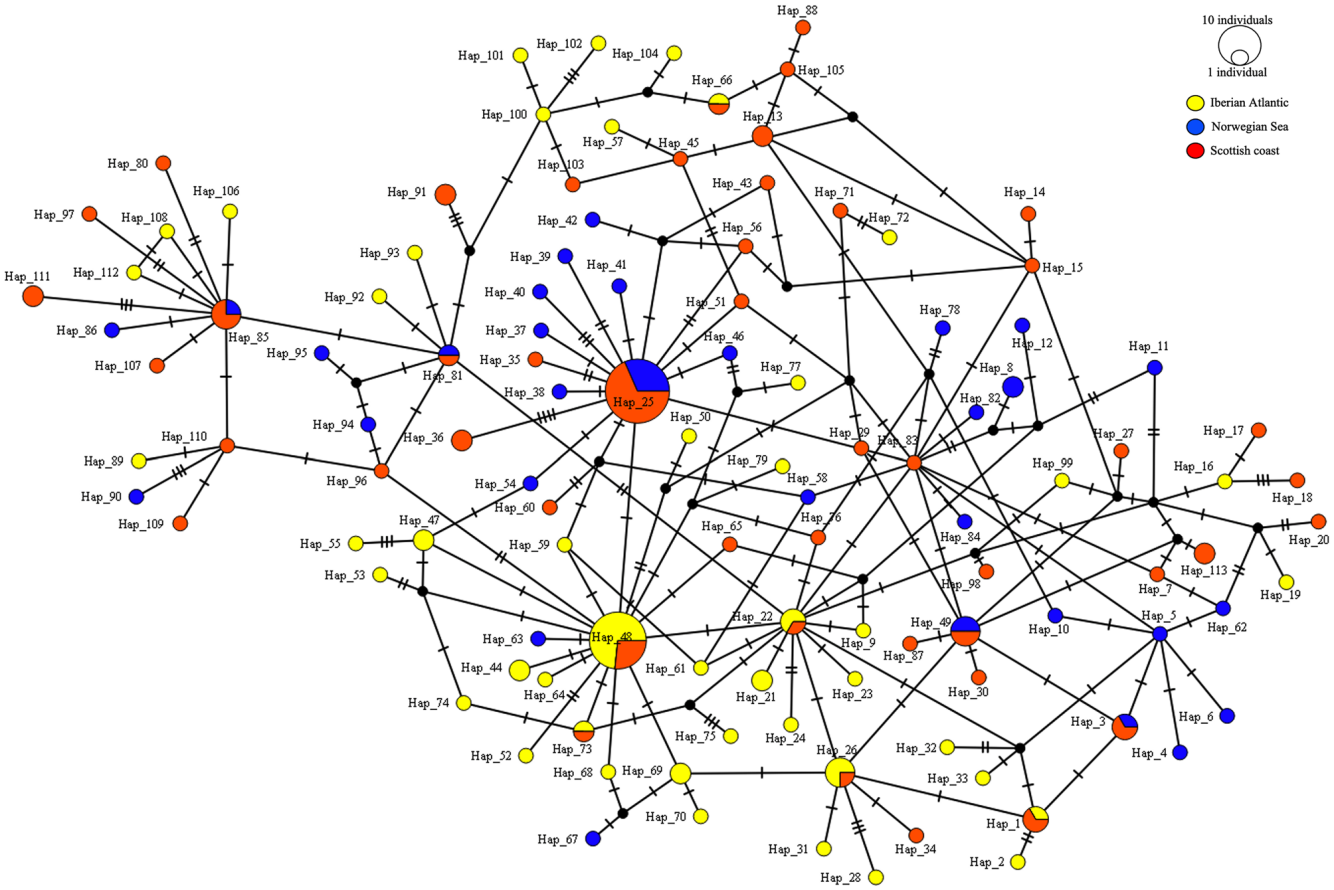
TCS network of *A. simplex* (s.s.) from cetaceans’ definitive hosts obtained using the *cox*2 sequences data set. Haplotypes (Hap1, Hap2, Hap3, etc.) are reported. Hatch marks indicate mutations. Circle sizes are proportional to the number of shared individuals per haplotypes.

The haplotype parsimony network (TCS) inferred from mtDNA *cox2* haplotypes of *A. simplex* (s.s.) (Fig. 4) shows that the presence of two major haplotypes, i.e. Hap25 and Hap48. Hap25 was shared by several *A. simplex* (s.s.) individuals collected in the cetacean hosts from the Norwegian Sea and Scottish coast of the NE Atlantic Ocean, whereas Hap48 was shared mostly by the *A. simplex* (s.s.) metapopulations collected in the Iberian and Scottish Atlantic (Fig. 4). The TCS analysis revealed the existence of several unique haplotypes in the Norwegian metapopulation of *A. simplex* (s.s.) and in Iberian Atlantic waters. Moreover, TCS also shows concordance between the haplotype clustering and geographic origin of the endoparasite samples (Fig. 4).

**Figure 5:**
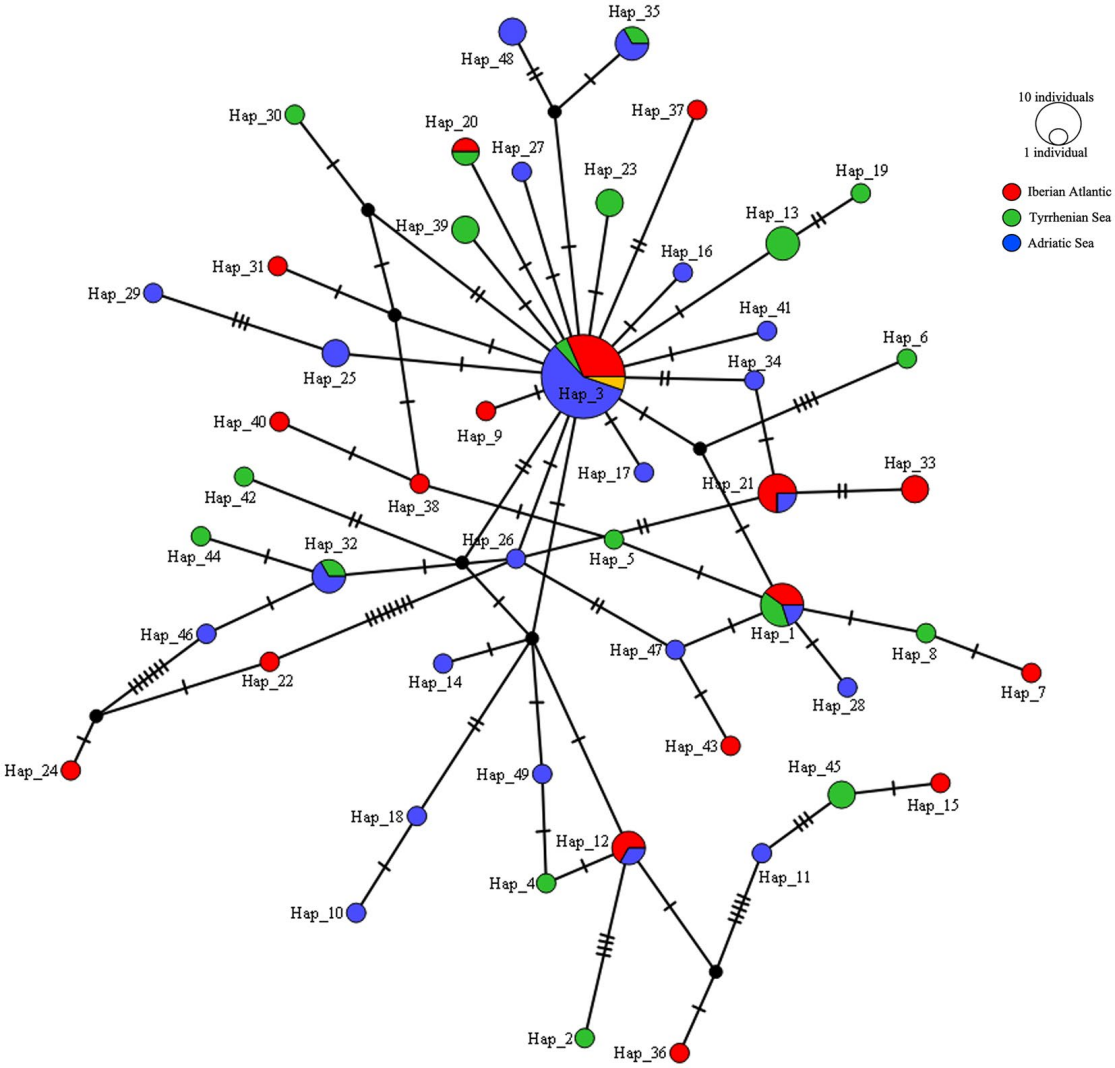
TCS network of *A. pegreffii* from cetaceans’ definitive hosts obtained using the *cox*2 sequences data set. Haplotypes (Hap1, Hap2, Hap3, etc.) are reported. Hatch marks indicate mutations. Circle sizes are proportional to the number of shared individuals per haplotypes.

TCS analysis of mtDNA *cox2* haplotypes of *A. pegreffii* resulted in a star-like tree (Fig. 5), with Hap3 being the most frequent haplotype, shared by all the metapopulations of the parasite (Fig. 5). A high frequency of certain unique haplotypes in the parasite specimens from the definitive hosts stranded on the Spanish Atlantic coast and from the Adriatic Sea was observed in *A. pegreffii* (Fig. 5).

**Figure 6:**
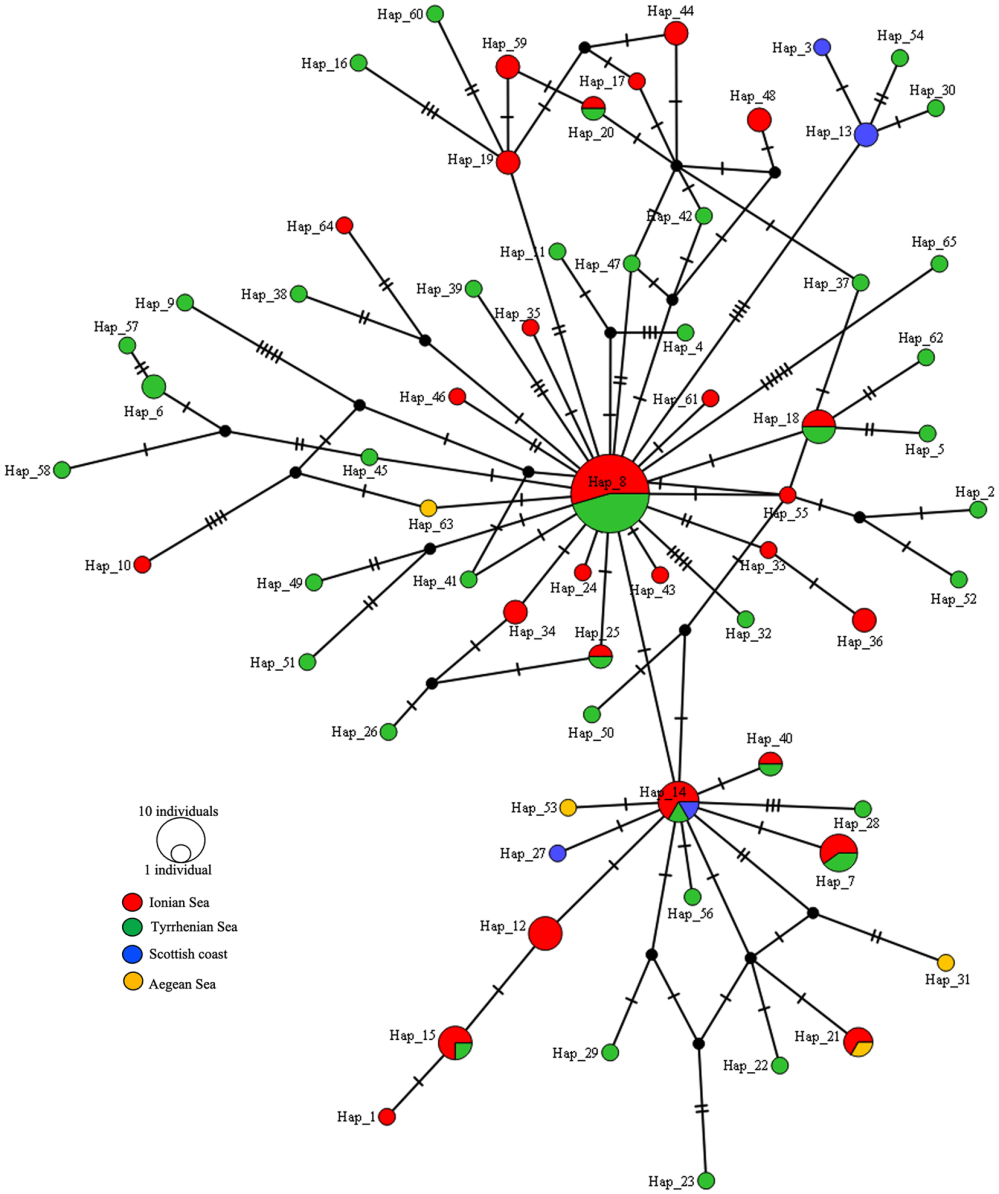
TCS network of *A. physeteris* from cetaceans’ definitive hosts obtained using the *cox*2 sequences data set. Haplotypes (Hap1, Hap2, Hap3, etc.) are reported. Hatch marks indicate mutations. Circle sizes are proportional to the number of shared individuals per haplotypes.

Finally, results from the TCS analysis of *A. physeteris* haplotypes did not show grouping by geographic regions (Fig. 6), but instead revealed a star-shaped pattern, with Hap8 (likely the ancestral haplotype) being the most frequent haplotype, shared by specimens from the Ionian Sea and the Tyrrhenian Sea. The analysis also showed the existence of many haplotypes in several areas of the Mediterranean Sea, differentiated from each other by a few substitutions (Fig. 6). The haplotype diversity (*h*) of *A. simplex* (s.s.), estimated by the analysis of mtDNA *cox2* sequences, was high, ranging between 0.97 and 0.98 in parasite metapopulations from Iberian Atlantic waters and the Norwegian Sea, respectively (Table 3). Slightly lower values were found in *A. pegreffii* (on average, *h* ≈ 0.95) and in *A. physeteris* (on average, *h* ≈ 0.95) (Table 3). The average value of nucleotide diversity within species was π ≈ 0.007, π ≈ 0.005 and π ≈ 0.006 in *A. simplex* (s.s.), *A. pegreffii* and, *A. physeteris*, respectively (Table 3).

**Table 3:** Genetic diversity of *A. simplex* (s.s.), *A. pegreffii* and *A. physeteris* from the cetaceans’ definitive host, by collection localities.

	N	Nh	π ± SD	Hd ± SD	K	S
***A. simplex* (s.s.)**						
Norwegian Sea	38	31	0.00731 ± 0.00065	0.976 ± 0.017	4.32717	36
Scotland coast	72	47	0.00774 ± 0.00053	0.963 ± 0.015	4.58412	43
Iberian Atlantic coast	62	46	0.00691 ± 0.00053	0.972 ± 0.014	4.09096	56
***A. pegreffii***						
Adriatic Sea	37	23	0.00592 ± 0.00086	0.911 ± 0.041	3.15616	30
Tyrrhenian Sea	30	20	0.00974 ± 0.00158	0.968 ± 0.017	5.20000	37
Iberian Atlantic coast	22	15	0.01019 ± 0.00148	0.961 ± 0.024	5.44156	22
***A. physeteris***						
Ionian Sea	55	26	0.00543 ± 0.00049	0.938 ± 0.021	2.99125	30
Tyrrhenian Sea	51	39	0.00861 ± 0.00081	0.962 ± 0.020	4.74275	71

Number of sequences analyzed (N), number of haplotypes (Nh), nucleotide diversity (per site) (π) and haplotype diversity (Hd) with their relative standard deviation (SD), average number of nucleotide differences (K) and number of polymorphic sites (S), are reported.

## Discussion

### Identification of *Anisakis* spp. in cetaceans

Knowledge of the occurrence and distribution of adult stage worms of the genus *Anisakis* in their definitive hosts is mostly based on opportunistic samples, obtained during necropsy investigations of cetaceans which were stranded, bycaught in fishing gear or taken by whaling^6,8,40^. Use of stranding records is a valid and sustainable approach to study the diversity of local marine mammal species^41–45^, and to investigate the parasite fauna hosted by these animals. Stranding events can occur for several reasons, including natural causes such as illness, environmental factors, and anthropogenic causes including bycatch, vessel strikes, plastic ingestion, or acoustic trauma^46–54^. Despite these advantages, parasitological studies on stranded cetaceans are affected by the decomposition state of the animals. It is also possible that animals in poor nutritional condition or moribund due to disease or trauma are unlikely to have been successfully foraging before death, which may affect the parasite burden demonstrated at necropsy.

**Figure 7:**
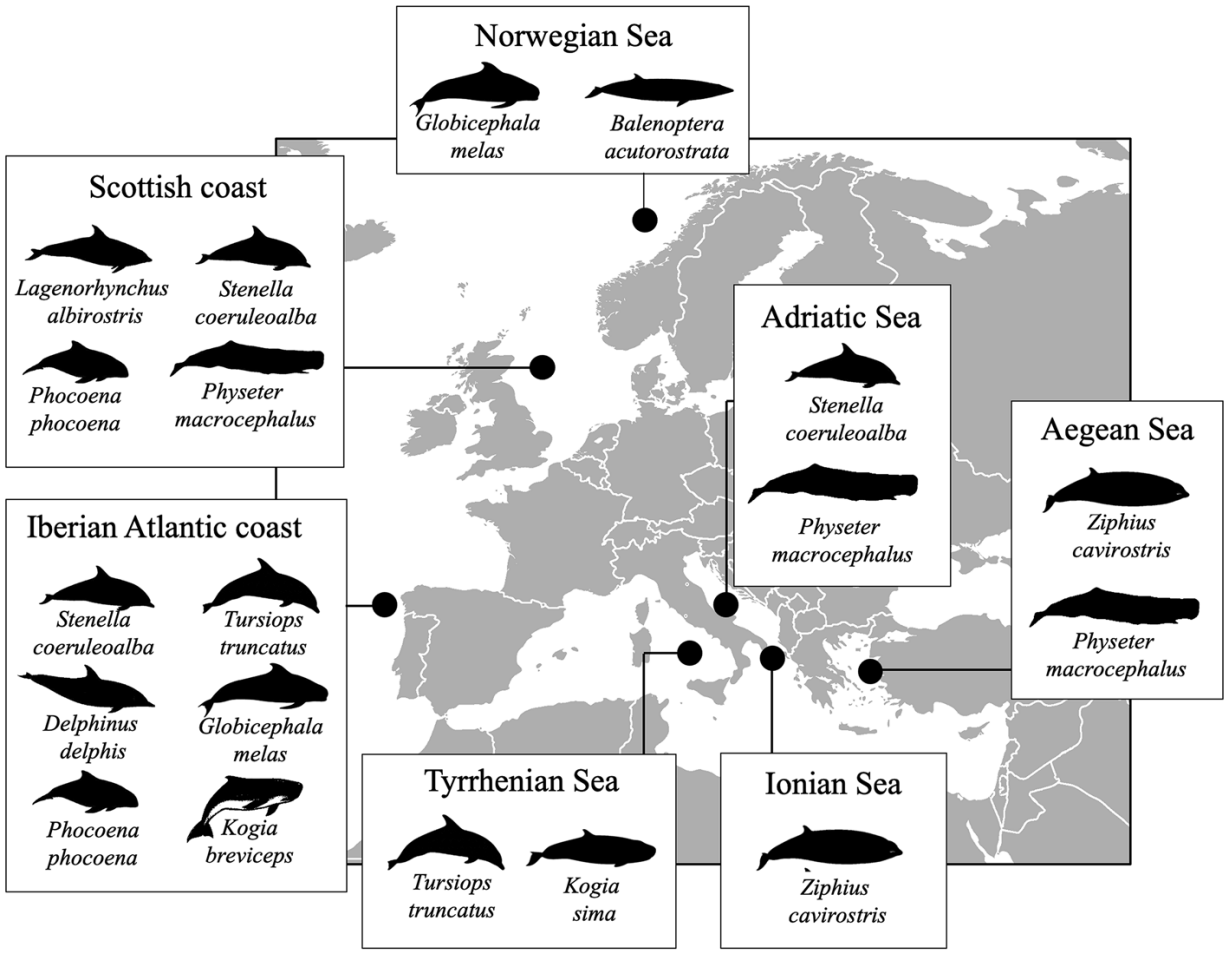
Sampling localities of *Anisakis* spp. in the Mediterranean Sea and NE Atlantic Ocean of the occasional cetacean strandings, which resulted in 34 specimens belonging to 11 species. The map was obtained from Wikimedia Commons, licensed-free (https://commons.wikimedia.org/wiki/File:BlankMap-Europe-v4.png), Roke was the unofficial uploader of the derivitive map CC BY-SA 3.0.

In this study, five *Anisakis* species, namely *A. simplex* (s.s.), *A. pegreffii, A. physeteris, A. brevispiculata* and *A. ziphidarum,* were detected from 11 species of cetacean stranded at various locations along the coasts of the NE Atlantic Ocean and Mediterranean Sea (Fig. 7, Table 4). In addition, a multilocus genotyping approach based on nuclear markers, applied to specimens of the sibling species *A. simplex* (s.s.) and *A. pegreffii* obtained from hosts occurring in a sympatric area (Iberian Atlantic waters), allowed the identification of two heterozygote genotypes, i.e. two F1 hybrids, showing a heterozygous pattern at all of the diagnostic nucleotide positions observed at the EF1 *α* − 1 nDNA and *nas* 10 nDNA loci (Suppl. Fig. 1). This finding suggests that these heterozygote genotypes most likely originated from a recent hybridization event between sympatric specimens of *A. simplex* (s.s.) and *A. pegreffii*. There have been reports of *A. simplex* (s.s.) and *A. pegreffii* L3 heterozygote genotypes in the same sympatric area from several fish species, including species which are likely to be eaten by the studied cetaceans^26,29,55–61^. Thus, hybridization events between species of the *A. simplex* (s. l.) complex are probably a common phenomenon in sympatric areas, as also recently reported from southern oceans based on a multinuclear genotyping approach^29^.

**Table 4:** Number (N_p_) of specimens of *Anisakis* spp. collected in the definitive host individuals (N_h_) from different sampling area, studied through the sequence analysis of the mtDNA *cox*2 and *EF*1*α *− 1 nDNA and the direct genotyping (ARMS) of *nas* 10 nDNA.

Host family	Cetacean species	N_h_	Sampling area	Coordinate	N_p_	N_p_ mtDNA *cox*2	N_p_ *EF*1*α *− 1 nDNA	N_p_ ARMS *nas* 10 nDNA
Balenopteridae	*Balenoptera acutorostrata*	1	Norwegian Sea	63°27′N–6°24′E	25	25	–	–
Physeteridae	*Physeter macrocephalus*	1	Scotland coast	58°19′N–8°17′W	7	7	–	–
		5	Adriatic Sea	42°04′N–14°46′E	60	60	–	–
		1	Aegean Sea	38°32′N–25°04′E	1	1	–	–
Kogiidae	*Kogia breviceps*	1	Iberian Atlantic coast	42°21′N–9°12′W	3	3	3	3
	*Kogia sima*	1	Tyrrhenian Sea	40°20′N–14°47′E	107	107	–	–
Ziphiidae	*Ziphius cavirostris*	1	Aegean Sea	38°32′N–25°04′E	4	4	–	–
		1	Ionian Sea	37°36′N–18°14′E	2	2	–	–
Delphinidae	*Delphinus delphis*	3	Iberian Atlantic coast	42°21′N–9°12′W	34	34	34	34
	*Lagenorhynchus albirostris*	1	Scotland coast	58°19′N–8°17′W	22	22	–	–
	*Stenella coeruleoalba*	1	Scotland coast	58°19′N–8°17′W	21	21	–	–
		3	Iberian Atlantic coast	42°21′N–9°12′W	22	22	22	22
		3	Adriatic Sea	42°18′N–15°00′E	44	44	–	–
	*Tursiops truncatus*	1	Iberian Atlantic coast	42°21′N–9°12′W	3	3	3	3
		2	Tyrrhenian Sea	40°20′N–14°47′E	6	6	–	–
	*Globicephala melas*	1	Faroe Islands	62°47′N–6°54′W	34	34	–	–
		2	Iberian Atlantic coast	42°21′N–9°12′W	27	27	27	27
Phocoenidae	*Phocoena phocoena*	1	Scotland coast	58°19′N–8°17′W	9	9	–	–
		3	Iberian Atlantic coast	42°21′N–9°12′W	47	47	47	47

The phylogenetic tree topology, based on the mtDNA *cox*2 sequences obtained (Fig. 3), differentiated *Anisakis* spp. into four well-defined clades, in accordance with previous studies^3,23^. The first clade comprised species of the *A. simplex* complex, i.e. *A. simplex* (s.s.), *A. pegreffii* (and *A. berlandi*, not found in the current study). The second clade included *A. ziphidarum* and *A. nascettii* (the latter not found in the current study), while the third clade comprised *A. physeteris, A. brevispiculata,* and *A. paggiae* (the latter not found in the current study).

### Host preference by *Anisakis* spp.

The well-known existence of a host specificity pattern in *A. simplex* (s.s.) and *A. pegreffii* for dolphins and baleen whales has been further supported by the present findings. Indeed, the three sibling species of the *A. simplex* (s. l.) complex are primarily parasites of the cetacean families Delphinidae, Monodontidae, Phocoenidae and Balaenopteridae^1,2,6,8,19,23,38,62^, parasitizing these hosts in different geographical areas, depending on the range of distribution of both host and parasite species^3,7,30^.

In the current study, adult nematodes of *A. simplex* (s.s.) and *A. pegreffii* were found in all the species of delphinids that were examined and in a minke whale specimen (Table 1, Figs. 1 and 2). Adult parasites of these two species can often be found in syntopy in the same definitive hosts in areas where the geographical range of the two parasite species overlaps^26,60^, as observed in bottlenose dolphins, striped dolphins, common dolphins and harbour porpoises inhabiting the waters off the Iberian coast (Table 1, Figs. 1 and 2). A sympatric and syntopic distribution of these two species was previously observed in other delphinids and whales sampled along the Japanese coasts, such as bottlenose dolphin, minke whale, and sei whale (*B. borealis*)^6^. Similarly, a syntopic occurrence of *A. pegreffii* and *A. berlandi* was reported in long-finned pilot whales from New Zealand^30^, as well as in a killer whale (*Orcinus orca*) from Argentinian waters^7^. Another relevant observation concerning the preference of these parasite species for certain definitive hosts is the different size reached by mature specimens of *A. simplex* (s.l.) in mysticetes, killer whales and smaller dolphins^6,8^. Gomes et al.^6^, and Ugland et al.^8^, reported that *A. simplex* (s.s.) and *A. pegreffii* specimens seem to reach bigger sizes in mysticetes (sei whales and minke whales) than in Delphinidae (bottlenose dolphins, long finned pilot whales and striped dolphins). Bigger whales probably offer a more beneficial microhabitat to these parasite species compared to smaller delphinids, enhancing their fitness potential. However, the coevolutionary implications related to these observations have not yet been clarified.

This investigation further confirms that cetaceans of the families Physeteridae (i.e. sperm whale) and Kogiidae (i.e. dwarf sperm whale*, K. sima*, and pygmy sperm whale, *K. breviceps*) are the main definitive hosts for the species included in clade 3 (Figs. 1, 2, 3), comprising *A. physeteris*, *A. brevispiculata* and *A. paggiae*. However, a very rare infection of (preadult) *A. simplex* (s.s.) and *A. pegreffii* was recorded in physeterids in the present study. Santoro et al.^63^ reported the presence of a few immature specimens of *A. pegreffii* in syntopy with fully developed adult specimens of *A. physeteris* located in the stomach of the same specimen of dwarf sperm whale examined in the present study*.* A rare case of infection with *A. simplex* (s.s.) in a pygmy sperm whale from the Caribbean Sea was also previously observed^19^, but the developmental stage of the worms was not reported. The rare cases of co-infection with both *A. simplex* (s.s.) and *A. pegreffii* in physeterid species, mostly at the preadult stage, strengthen the hypothesis that infections with these two parasite species in physeterids are quite uncommon and probably accidental. In this study, *A. physeteris* was found to be the most common anisakid in the sperm whale, representing 97% of the identified specimens, and 82% of the anisakids recovered from dwarf sperm whale. These proportions are similar to those previously reported in other studies^39,64^.

The host preference of *A. physeteris* for odontocetes belonging to the family Physeteridae, specifically the sperm whale, could be related to the ecology and the peculiar life cycle of this *Anisakis* species. *A. physeteris* has been found sporadically in fish species, with low infection rates^3,34,35^ probably because it relies on deep-sea squid species as transport/paratenic hosts^65^. Large, deep-dwelling cephalopods may act as reservoir hosts for this anisakid species, and may thus be crucial to complete its life cycle and maintain its distribution. In fact, the whole life cycle of this parasite species is probably strictly connected to deep sea ecosystems. Considering the feeding behaviour of the sperm whales, the role of squid of the family Histiotheutidae could be crucial in the life cycle of *A. physeteris*, as recently suggested by the finding of *A. physeteris* larvae in *Histioteuthis bonnellii* in the depths of the Central Mediterranean Sea (Tyrrhenian Sea)^65^. Thus, deep-dwelling squid species, which are common prey of physeterid whales^39,43,66–69^, could be a suitable paratenic/transport host for this *Anisakis* species. The stomachs of three out of the six sperm whales stranded along the Italian coast of the Adriatic Sea in 2017 showed a mean abundance of 706 specimens of *A. physeteris per* whale^39^. In the same stomachs, along with this burden of preadult and adult specimens of *A. physeteris*, over 4000 cephalopods beaks were counted, for each whale. Of these beaks, 95% were classified as belonging to histioteuthids (71% *H. bonnellii* and 24% *H. reversa*), while the rest were *Ancistrocheirus lesueurii* (Ancistrocheiridae) and *Octopoteuthis sicula* (Octopoteuthidae)^39^. The co-occurrence of pre-adults and adults of *A. physeteris* along with large numbers of squid beaks in the above-mentioned stomachs indicates a possible link between these deep-sea squid species, probably carrying *A. physeteris* parasites in high numbers. A similar finding was reported from a sperm whale stranded along the Southeastern coast of Italy, which was parasitized by several hundred adult *A. physeteris*^70^ and had the stomach full of beaks of the squids *H. bonnellii* and *Ancistroteuthis lenchisteini* (Mattiucci and Cipriani pers. obs.).

It could be hypothesized that *A. pegreffii* and/or *A. simplex* (s.s.) were ingested along with *A. physeteris.* The latter*,* being adapted to the specific stomach-microhabitat of physeterid hosts, could be more competitive. Similar dynamics have been observed in several other sibling species of anisakid nematodes^71^ as well as in other helminth parasites^72^.

Finally, two *A. ziphidarum* adult specimens, identified from a Cuvier´s beaked whale (*Ziphius cavirostris*) stranded on the Italian coast of the Ionian Sea, clustered in Clade 2 (Fig. 3), along with the closely related species *A. nascettii.* To date, *A ziphidarum* is the only anisakid species found in Cuvier's beaked whale from the Mediterranean Sea^73^ while *A. nascettii* (not found in the current study) was mainly reported from beaked whales of the genus *Mesoplodon*^4,74^. The feeding habits of beaked whales suggest that the life cycles of *A. ziphidarum* and *A. nascettii* may mostly involve deep-sea intermediate hosts such as deep-sea squids and mesopelagic fish. Indeed, ziphiids commonly prey on squid belonging to the families Onychoteuthidae and Histiotheuthidae, rather than fish^75–77^.

The association observed between *Anisakis* spp. and several cetacean taxa may reflect a co-evolutionary history between these endoparasites and their hosts over the millennia, driven by common trophic adaptations. In particular, the distinct clades formed by the species of *Anisakis* have been suggested to “mirror” the clades so far reported in the phylogenetic analysis of their main definitive hosts^2,21^.

### Drivers of the distribution of *Anisakis* spp. in cetacean species of European waters

Among the drivers that shape the geographical range of the various *Anisakis* species, particular consideration should be given to a dispersal mechanism involving both their definitive (cetaceans) and intermediate/paratenic (fish and squid) hosts.

The infection pattern of the adult *Anisakis* specimens recorded in their final hosts (Fig. 1) was largely in accordance with their documented distribution in intermediate/paratenic hosts from the Atlantic Ocean and the Mediterranean Sea^3,35^.

Generally, in the NE Atlantic Ocean, *A. simplex* (s.s.) and *A. pegreffii* are distributed along a latitudinal gradient^1,3^. In this study, we confirmed that *A. pegreffii* is the most widespread parasite species occurring in cetaceans from the Mediterranean Sea. In the NE Atlantic Ocean, the prevalence of *A. pegreffii* progressively declines northwards along the NorthWest Spanish coast (Fig. 1) and is absent from hosts from northern waters. An opposite geographic trend was found in *A. simplex* (s.s.), which showed a south-to-north increase in abundance when in sympatry with *A. pegreffii* in cetaceans stranded on the coast of Spanish Galicia, and was the only *Anisakis* species present in the cetaceans from the NE Atlantic Ocean (Fig. 1). *A. simplex* (s.s.) is the only *Anisakis* species so far reported from cetaceans in the northern NE Atlantic Ocean. This is confirmed by a recent study on *Anisakis* spp. in stranded harbour porpoises from the Norwegian Sea whose were identified molecularly as individuals of *A. simplex* (s.s.)^15^. In the same host, a study on specimens stranded on the North Sea, Baltic Sea and North Atlantic coasts, detected the presence of *A. simplex* (s.s.), as well as the presence of accidental parasites, e.g. *Pseudoterranova decipiens* (s.s.) (usually associated with pinnipeds as definitive hosts) and *Hysterothylacium aduncum* (a parasite maturing in teleost fish)^78^. *Anisakis* sp. was previously detected in 24 minke whales from the NE Atlantic Ocean, from which a large number of nematodes was recovered during whaling operations in the Barents Sea^8^. The worms were not molecularly identified at species level but presumably consisted of *A. simplex* (s.s.)^23^. A high prevalence of infection with adults of *Anisakis* in long-finned pilot whales was also reported from Faroe Islands^8,79^, even if identification was not provided. Mattiucci et al.^23^ identified several adults of *A. simplex* (s.s.) obtained from this same host in the Norwegian Sea.

In the study of genetic architecture drivers of *Anisakis* spp., the high dispersal capacity of some definitive and intermediate/paratenic hosts species (mainly pelagic fish) has a crucial role in maintaining gene flow between parasite populations. Indeed, some cetacean hosts are capable of remarkable migrations to feed or mate, carrying their parasites with them. However, it should be considered that larval *Anisakis* specimens in highly migratory fish species may have even a wider dispersal potential, since the larvae may remain latent for years^9,80^. Larval *Anisakis* spp. can live for years in their transport hosts^9,80^, while they probably need around 40–60 days to mature in their definitive hosts^8,81^, Mattiucci pers. obs. from in vitro culture experiments]. Highly migratory hosts, particularly in periods of intense migrations (mating or feeding), can spread their parasites over broad spatial scales, dispersing specimens even out of the geographical range in which they could find favourable ecological conditions for completing their life cycle. These migrating fish could be predated by suitable definitive hosts in areas far away from the original infection, where abiotic conditions, depending on their suitability for completion of the parasite life cycle, would represent a limiting factor for parasite dispersal.

For instance, *A. simplex* (s.s.) specimens have been rarely detected in the Mediterranean Sea. The few reports available^34,35^, of larval stages found in fish, refer mainly to the Alboran Sea, which is considered ecologically more similar to the Atlantic waters than to the rest of the Mediterranean Sea. However, three adult specimens of *A. simplex* (s.s.) were identified by sequences analyses of the ITS region of rDNA and mtDNA *cox*2 from bottlenose dolphin and striped dolphin*,* as well as two larval specimens from bluefin tuna (*Thunnus thynnus*) in the Adriatic Sea^28,38,82,83^. The presence of *A. simplex* (s.s.) outside the usual limits of its geographical range may be related to the wide migratory capability of the intermediate and definitive host species in which the parasite has been recorded. Hence, these cetaceans and pelagic fish species could have acquired the infection in areas within the usual range of *A. simplex* (s.s.) and these findings may not be indicative of the “true” habitat where these species could complete their life cycle.

A similar hypothesis could explain the rare findings of larval *A. pegreffii* in fish from the northern waters of the NE Atlantic Ocean, although they have not so far been found as adults in cetacean hosts from the same waters. Larvae of *A. pegreffii* were detected in the highly migratory Atlantic mackerel (*Scomber scombrus*) caught in the Norwegian Sea^84^, also in Atlantic cod (*Gadus morhua*) from the North Sea^85^, the latter known for being an active predator of migratory prey (such as Atlantic mackerel and Atlantic herring *Clupea harengus*). The biotic and/or abiotic conditions of northern waters may represent important limiting factors for the completion of the life cycle of this parasite in this geographical area. Among the drivers of the geographical range of *Anisakis* spp., certain abiotic conditions (i.e. temperature or salinity) may be considered limiting factors to the dispersal and range expansion of this parasite species, probably impeding the egg survival or the first developmental stages of hatched larvae to succeed in finding a suitable host in some oceanographic areas.

The combined “matrix” of the many definitive, intermediate and paratenic hosts of *Anisakis* spp. comprises a complex heterogeneous and constantly shifting mosaic, potentially influencing the endoparasite population structure and demography at both local and wider levels. As is the case for many other pelagic marine organisms, cetaceans are highly mobile, their movements driven by prey availability and suitable conditions for their reproduction^42,86–88^. The long-range dispersal of certain cetacean species, along with the dispersal contribution provided by highly migratory fish hosts, may maintain high levels of gene flow between *Anisakis* populations throughout their distribution range. However, cetaceans often show cryptic population genetic structure over a small geographical range, much more than expected for such highly migratory marine mammals^89^. Some genetic sub-structuring has been found in both *A. simplex* (s.s.) and *A. pegreffii*, despite the general high gene flow values observed between populations of these species^2^. This observation is supported by the haplotype network analysis provided in the present study (Fig. 4), showing a certain geographic separation in *A. simplex* (s.s.) individuals, with a significant differentiation between the parasites collected from cetaceans of the Norwegian Sea compared to conspecifics from definitive hosts of the NE Atlantic Ocean (on average, *F*_*st*_ ≈ 0.08) (Table 2). Cetacean population units with different feeding ecology^90^ and/or range of distribution may be responsible for maintaining the genetic differentiation observed in *A. simplex* (s.s.). Indeed, while a weakly significant genetic substructure of the parasite species appears to exist among metapopulations sampled from several definitive hosts, this structure seems to be stronger when analysing the parasite's population by geographic area of the host species. In turn, this seems to suggest that the geographic origin of the host species could be major driver explaining the sub-structuring of the parasite populations.

Interestingly, in a previous population genetic study of *A. simplex* (s.s.) larvae in herring from several NE Atlantic fishing grounds based on mtDNA *cox*2*,* the haplotype diversity values were, on average, in the same range as those observed in the present study^91^. Furthermore, the population showing the highest differentiation was the one from the most northern area, specifically the Norwegian Sea^91^. As herring is an important food source for cetacean species, such as minke whales, killer whales, and humpback whales (*Megaptera novaeangliae*), it is reasonable to expect that the Norwegian *A. simplex* (s.s.) population detected in herring overlaps with the one identified here based on network and haplotype analysis conducted on the corresponding adult stage in cetacean predators.

Moving to the southernmost waters of the NE Atlantic, significant genetic differentiation was observed (on average *F*_*st*_ ≈ 0.05) between parasite populations of *A. pegreffii* from dolphins in the Mediterranean Sea and Iberian Atlantic waters investigated in this study. Significant genetic differentiation was reported between populations of the short-beaked common dolphin from the Mediterranean Sea and adjacent Atlantic waters (Galicia and Portugal), reflecting a differential distribution and habitat use for populations of this dolphin species^92^. This finding suggests that the populations sub-structuring of the endoparasites (i.e. *A. pegreffii*) along their distribution range may overlap with different cetacean populations from different European waters.

Regarding the population genetics of *A. physeteris* identified in sperm whales stranded along the coast of the southern Adriatic Sea and the Tyrrhenian Sea, the TCS analysis has shown a star-like shape with many haplotypes (Fig. 6), most of which are shared by all the nematodes sampled in the same host from different geographical areas. However, a weak but significant level (*p* = 0.03) of population substructuring (on average *F*_*st*_ ≈ 0.01) was found between the metapopulation of *A. physeteris* collected from sperm whales stranded on the coast of Vasto (animals which died on the Southern Adriatic Sea coast but which come from the deeper waters of the Ionian area of the Mediterranean Sea^39,93^) and those from the dwarf sperm whale stranded along the Tyrrhenian Sea coast. A consistent level of differentiation (on average, *F*_*st*_ ≈ 0.18, *p* = 0.003) was observed between this parasite species recovered from the sperm whale from Scottish waters (Atlantic Ocean) and those from the Mediterranean Sea (data not shown). Although these preliminary findings need to be validated by examining a higher number of individuals from the NE Atlantic, they appear to reflect the Mediterranean Sea and the Atlantic Ocean sub-structuring observed in the main host of *A. physeteris*, the sperm whale. In fact, the genetic analysis conducted on whales stranded on the coast of the Adriatic Sea in two separate events (Varano in 2009 and Vasto in 2014) showed that all the specimens belonged to a unique haplotype, similar to other individuals from the Mediterranean Sea^39,93,94^, but genetically distinct from the adjacent populations of the Atlantic Ocean^95^.

Overall, these observations suggest that the genetic structure, the phylogeography and the ecology of the definitive hosts species are likely to also shape the genetic structure of their anisakid nematodes, as has been recently observed in other nematode parasites of marine mammals, such as the pinniped parasite *Uncinaria lucasi*^96^.

## Conclusions

Studies on the adult stage of nematode parasites of the genus *Anisakis* mostly rely on occasional stranding events of their definitive hosts, making sampling unpredictable and opportunistic. At the same time, the scientific data obtained are crucial to understand the biology, host specificity, and life cycle of the *Anisakis* species. In fact, the ecology, evolutionary history, geographical distribution, and migration of cetaceans are among the drivers defining their parasites’ geographic range and population genetic structure. Drivers of marine speciation are rarely obvious, because the distributions of marine organisms (and changes in these distributions) tend to be poorly documented, implying a high level of uncertainty, and also because the oceans are continuous environment with few geographical barriers. Therefore, we have little understanding of the nature and mechanisms of reproductive isolation in the marine environment that determine the host-parasite association between *Anisakis* spp. and their cetacean hosts. This study indicates a congruence between the geographic distribution of the adult stages of the *Anisakis* species in their final hosts and the larval stages in their fish hosts. While *Anisakis* spp. are generalists for paratenic/transport hosts, the findings on definitive host-parasite association at the adult stage suggests instead a strong preference by some *Anisakis* spp. for certain cetacean species or families. This host preference is likely to be driven by coevolutionary aspects and mediated by definitive host feeding ecology and geographic distribution. Novel data on genetic structuring, geographical distribution, and relative proportions of different parasites species in their hosts can help in unravelling the complex life cycles of these parasites. Alongside the influence of abiotic and biotic limiting factors, the intermediate, paratenic and definitive hosts, according to their ecological characteristics, all contribute to shape the current distributions of *Anisakis* spp. in the world’s oceans.

## Methods

### Cetacean parasites sampling data

Samples of *Anisakis* spp. were collected from 34 individuals belonging to 11 cetacean species stranded in locations along the Mediterranean Sea and NE Atlantic Ocean coast, between 2004 and 2019 (see Table 4 and Fig. 7). All cetacean individuals were necropsied following a standardised method^97^. All parasites were recovered from the stomach chambers of their hosts, usually during necropsy, and generally it was possible to obtain only a random subsample of the *Anisakis* spp. specimens infecting in the host. On a few occasions it was possible to count or estimate the total burden of parasites infecting the host.

In total, N = 478 anisakid nematodes were identified to genus level based on morphological characters reviewed by Mattiucci et al.^3^. Mature adults were separated from pre-adults, based on the visibility in the former of caudal papillae and spicules in males and eggs in females). The sampled nematodes were stored frozen at − 20 °C, or in 70% ethanol, for further molecular analyses.

### Genetic identification of anisakid parasites

The nematode specimens were identified by sequence analysis of the mitochondrial cytochrome *c* oxidase II (mtDNA *cox*2) gene locus^23,25^. Specimens of *A. pegreffii* and *A. simplex* (s.s.) (N = 134), as inferred from the mitochondrial marker, collected from cetaceans stranded in sympatric areas for these two sibling species, were also identified using nuclear markers. In particular, sequence analysis of the diagnostic gene locus elongation factor EF1 *α *− 1 of nDNA^26^ and direct genotyping determination of the diagnostic nuclear metallopeptidase *nas*10 locus by ARMS-PCR^27^ were performed on these 134 specimens. In addition, sequence analysis of the same gene locus (*nas* 10 nDNA) was carried out on those individuals which showed a heterozygote pattern.

Total DNA was extracted from ~ 2 mg of tissue from each specimen by using the Quick-gDNA Miniprep Kit (ZYMO RESEARCH), following the manufacturer’s instructions. For sequencing of the *cox*2 gene, polymerase chain reaction (PCR) amplification was performed using the primers 211F (5′-TTTTCTAGTTATATAGATTGRTTTYAT-3′) and 210R (5′-CACCAACTCTTAAAATTA TC-3′)^98^. PCR was carried out according to the procedures described by Mattiucci et al.^23^.

The EF1 *α* − 1 nDNA gene was amplified using the primers EF-F (5′-TCCTCAAGCGTTGTTATCTGTT-3′) and EF-R (5′-AGTTTTGCCACTAGCGGTTCC-3′)^26^. PCR conditions and procedures followed those reported by Mattiucci et al.^26^. The EF1 *α − *1 nDNA sequences were compared with those previously deposited in GenBank, at the diagnostic positions (i.e., 186 and 286), as previously detailed by Mattiucci et al.^26^.

For sequencing the *nas*10 gene, PCR amplification was performed using the primers nas10F (5′-GATGTTCCTGCAAGTGATTG-3′) and nas10R (5′-CGCTATTAAGAGAGGGATCG-3′)^27^. PCR was carried out according to the procedures provided by Palomba et al.^27^. A direct genotyping determination of the nuclear *nas*10 locus was performed by ARMS-PCR at the gene locus *nas*10 by the combined use of OUT-F1 (5′-TATGGCAAATATTATTATCGTA-3′), OUT-R1 (5′-TATTTCCGACAGCAAACAA-3′), INN-F1 (5′-GCATTGTACACTTCGTATATT-3′), INN-R1 (5′-ATTTCTYCAGCAATCGTAAG-3′), following the procedures reported in Palomba et al.^27^. The PCR products were separated by electrophoresis using agarose gel (1.5%) stained with GelRed; 3 µL of the amplification products were visualized. The distinct banding patterns were detected using ultraviolet transillumination^27^.

The sequences obtained at the mtDNA *cox*2 locus were aligned with other *Anisakis* spp. sequences obtained in previous studies and deposited in GenBank, using ClustalX v2.0^99^. Alignments were manually edited and trimmed with BioEdit v7.0.5.3^100^, resulting in 580 characters and 9 taxa.

### Phylogenetic analysis inferred from results for the mtDNA *cox*2 gene locus

A phylogenetic tree of the *cox*2 mtDNA gene locus was constructed using Bayesian Markov Chain Monte Carlo (MCMC) analysis in BEAST v1.10.4^101^. The best-fit substitution model was selected using the Akaike information criterion (AIC) as implemented in jModeltest v2.1.7.^102^. GTR + I + G substitution model, strict molecular clock^103^ and a Yule speciation process were used as the tree priors^104^, with default parameters. A run of 10 million iterations was completed, logging parameter values after every 10,000 iterations, and checking for stationarity and effective sample sizes (ESS) from all the available parameters (cut-off > 200) with Tracer v1.7^105^. The first 10% of trees were discarded as burn in, and the remaining 9000 were analysed and visualized using TreeAnnotator v.1.10.4^101^, and FigTree v1.4.2 (http://tree.bio.ed.ac.uk/software/figtree/), respectively. *Toxocara canis* (AP017701) and *Ascaris suum* (X54253) were used as outgroups.

### Intraspecific genetic diversity of *Anisakis* spp. inferred from results at the mtDNA *cox*2 gene locus

The mean pairwise differences, among and within populations of *A. simplex* (s.s.), *A. pegreffii* and *A. physeteris* from the different cetacean samples were calculated from mtDNA *cox*2 sequences. Spatial analysis of molecular variance (AMOVA), conducted with ARLEQUIN version 3.5, with 1000 permutations, was applied to the genetic data sets obtained for the populations of *A. simplex* (s.s.), *A. pegreffii* and *A. physeteris*. The *F*_*st*_^106^ values were estimated by ARLEQUIN V3.5^107^ at the intraspecific level, among and within samples of *A. simplex* (s.s.), *A. pegreffii* and *A. physeteris* from different sampling areas. The *F*_*st*_ value ranges from a maximum of 1, indicating complete differentiation among sequences of pre-defined populations of the same *Anisakis* species collected from different regions to a value equal to a minimum of 0, which indicates no differentiation among the populations. Pairwise comparisons of *F*_*st*_ (assuming that *p* < 0.05 indicates a significant difference) were based on 1000 permutations of the data matrix. Intraspecific population genetic diversity of *A. simplex* (s.s.), *A. pegreffii* and *A. physeteris* among sampling areas was estimate based on the following standard statistical parameters: number of haplotypes (*Nh*), number of unique haplotypes (*Nuh*), nucleotide diversity (*π*), haplotype diversity (*Hd*), average number of differences (*K*), and number of polymorphic sites (*S*), using DnaSP V5.10.01 (http://www.ub.edu/dnasp)^108^. Haplotype network construction was carried out using PopART V1.7 (http://popart.otago.ac.nz/index.shtml)^109^. The analysis was performed using the statistical parsimony procedure (95% parsimony connection limit), implemented in TCS V3.5.1.2^110^.

## Supplementary Information

Supplementary Information 1.

Supplementary Information 2.

Supplementary Information 3.

## Data Availability

All data generated or analyzed during this study are included in this published article. The DNA sequences of the *Anisakis* species identified were deposited in the public sequence repository GenBank (NCBI National Center for Biotechnology Information—https://www.ncbi.nlm.nih.gov/genbank) [accession numbers: OM142467-81, OM210033-42].
