# Supplementary figures and images for: Distribution and genetic diversity of Anisakis spp. in cetaceans from the Northeast Atlantic Ocean and the Mediterranean Sea
Source: Sci Rep. 2022 Aug 11;12:13664. doi: 10.1038/s41598-022-17710-1 (PMC9372146; doi:10.1038/s41598-022-17710-1)

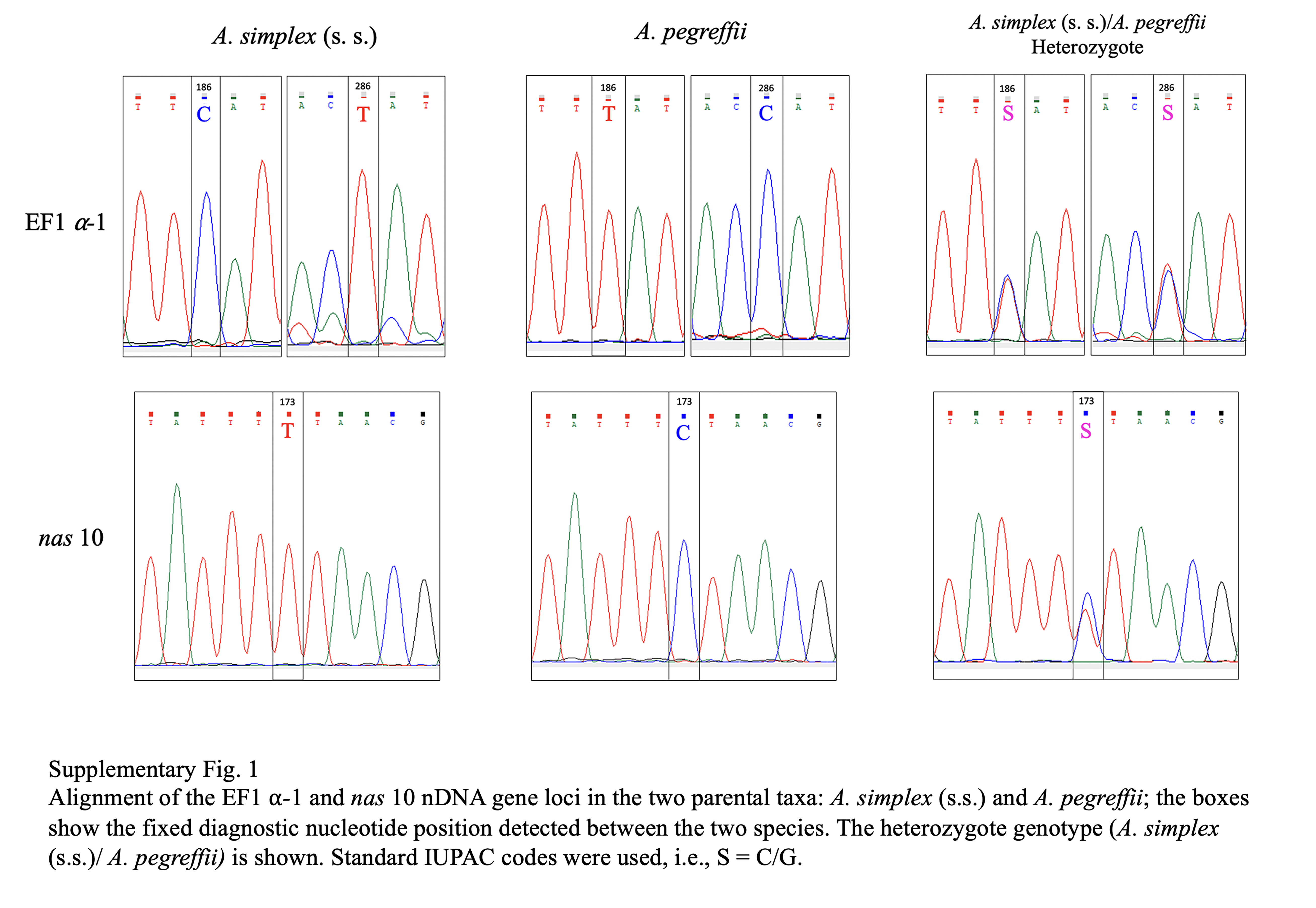

Supplement: Supplementary file 1 — Supplementary Information 1. [file 41598_2022_17710_MOESM1_ESM.tif]

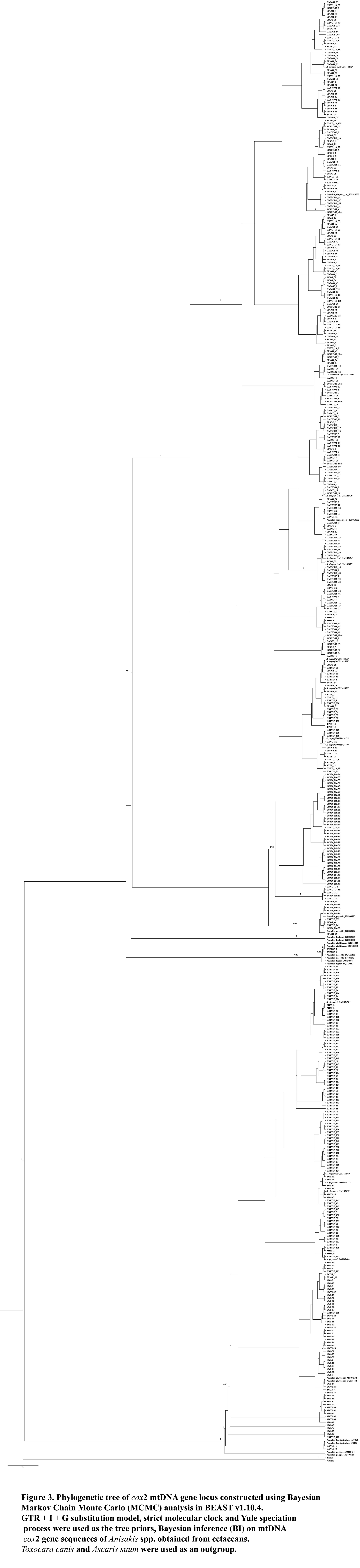

Supplement: Supplementary file 2 — Supplementary Information 2. [file 41598_2022_17710_MOESM2_ESM.tif]
