# Supplementary material for: Distribution and genetic diversity of Anisakis spp. in cetaceans from the Northeast Atlantic Ocean and the Mediterranean Sea
Source: Sci Rep. 2022 Aug 11;12:13664. doi: 10.1038/s41598-022-17710-1 (PMC9372146; doi:10.1038/s41598-022-17710-1)
Supplement: Supplementary file 3 — Supplementary Information 3. [file 41598_2022_17710_MOESM3_ESM.doc]

Suppl. Table 1. Analysis of molecular variance (AMOVA) between and within groups, of the species *A. pegreffii* (a), *A. simplex* (s.s.) (b) and *A. physeteris* (c).

| Source of variation |  | d.f. |  | Sum of squares |  | Variance components |  | Percentage variation |
| --- | --- | --- | --- | --- | --- | --- | --- | --- |
| a. *A. pegreffii* |  |  |  |  |  |  |  |  |
| Among populations |  | 3 |  | 9.733 |  | 0.056 |  | 2.52 |
| Within populations |  | 81 |  | 177.467 |  | 2.190 |  | 97.48 |
| Total |  | 84 |  | 187.200 |  | 2.247 |  |  |
|  |  |  |  |  |  |  |  |  |
| b. *A. simplex* (s.s.) |  |  |  |  |  |  |  |  |
| Among populations |  | 2 |  | 22.961 |  | 0.167 |  | 7.22 |
| Within populations |  | 169 |  | 364.289 |  | 2.155 |  | 92.77 |
| Total |  | 171 |  | 387.250 |  | 2.323 |  |  |
|  |  |  |  |  |  |  |  |  |
| c. *A. physeteris* |  |  |  |  |  |  |  |  |
| Among populations |  | 2 |  | 10.665 |  | 0.109 |  | 5.38 |
| Within populations |  | 112 |  | 216.440 |  | 1.932 |  | 94.62 |
| Total |  | 114 |  | 227.104 |  | 2.042 |  |  |
